# How Many Hospital Beds Are Required to Train a Nurse?

**Published:** 1920-12-18

**Authors:** 


### How Many Hospital Beds are Required to Train a Nurse?

The capacity of the training-schools to turn out nurses has, so far as we know, never been tested by the number of the beds. Our contemporary, the *New Zealand Journal of Health and Hospitals*, quoting with approval a passage from our own columns goes on to make an instructive calculation: "The increases in the number of hospital beds in New Zealand training-schools has been 1,279 during the past five years and the approximate increase in the number of nurses trained and registered 120. . . . At two and a-half beds to a nurse and three years' training it would appear to require over seven occupied beds to register one nurse annually. As a matter of fact, however, it takes about fifteen occupied beds to train a nurse, in actual practice, and a hospital of 375 beds seldom trains more than twenty-five a year, while in some cases the number registered is only about one to twenty-five beds." The bearing of such calculations on the increased number of probationers required under the eight-hour system should be pressed on public attention. It does, indeed, cost more to run a hospital on the eight-hour system, but then the value of that part of their work which consists in training nurses for the public service is largely increased. Even in schools where no rigid eight-hour system is attempted, more probationers are now being trained, and the full training capacity of all institutions is being utilised as never before.

